# SubTap, a Versatile 3D Printed Platform for Eavesdropping on Extracellular Interactions

**DOI:** 10.1128/mSystems.00902-21

**Published:** 2021-08-24

**Authors:** Caroline M. C. Birer-Williams, Rosalie K. Chu, Christopher R. Anderton, Erik S. Wright

**Affiliations:** a Biomolécules et Biotechnologies Végétales (BBV) EA 2106, Université de Tours, Tours, France; b Department of Biomedical Informatics, University of Pittsburgh School of Medicine, Pittsburgh, Pennsylvania, USA; c Environmental Molecular Sciences Laboratory, Pacific Northwest National Laboratory, Richland, Washington, USA; UiT - The Arctic University of Norway

**Keywords:** 3D printing, chemical ecology, exometabolome, intercellular communication, metabolomics, microbiome

## Abstract

Communication within the microbiome occurs through an immense diversity of small molecules. Capturing these microbial interactions is a significant challenge due to the complexity of the exometabolome and its sensitivity to environmental stimuli. Traditional methods for acquiring exometabolomic data from interacting microorganisms are limited by their low throughput or lack of sampling depth. To address this challenge, we introduce subtapping (short for substrate tapping), a technique for tapping into extracellular metabolites that are being transferred through the growth substrate during coculture. High-throughput subtapping is made possible by a new coculturing platform, named SubTap, that we engineered to resemble a 96-well plate. The three-dimensional (3D) printed SubTap platform captures the exometabolome in an agar compartment that connects physically separated growth chambers, which permits cell growth without competition for space. We show how SubTap facilitates replicable and quick detection of exometabolites via direct infusion mass spectrometry analysis. Using bacterial isolates from the soil, we apply SubTap to characterize the effects of growth medium, growth duration, and mixed versus unmixed coculturing on the exometabolome. Finally, we demonstrate SubTap’s versatility by interrogating microbial interactions in multicultures with up to four strains.

**IMPORTANCE** Improvements in experimental techniques and instrumentation have led to the discovery that the microbiome plays an essential role in human and environmental health. Nevertheless, there remain major impediments to conducting large-scale interrogations of the microbiome in a high-throughput manner, particularly in the field of exometabolomics. Existing methods to coculture microorganisms and interrogate their interactions are labor-intensive and low throughput. This inspired us to develop a solution for coculturing that was (i) open source, (ii) inexpensive, (iii) scalable, (iv) customizable, and (v) compatible with existing mass spectrometry instrumentation. Here, we present SubTap—a 3D printed coculturing platform that permits tapping directly into the growth substrate between physically separated, but interconnected, growth compartments. SubTap allows multiculture (with up to four distinct growth compartments) in spatially mixed or unmixed configurations and enables repeatable results with mass spectrometry, as shown by our validation with known compounds and cultures of one to four organisms.

## INTRODUCTION

Cells live in dense communities and interact through a diverse repertoire of small molecules. Capturing these extracellular metabolites (i.e., the exometabolome) by mass spectrometry provides a window into how cells transform and respond to their environment (1). Exometabolomics has been used to disentangle networks of interactions among cells from environments spanning the soil (2), human gut (3), and ocean (4). Furthermore, exometabolomics has been applied to optimize fermentation (5) and monitor contaminants (6) in industrial processes. Exometabolomes are challenging to study because of their immense diversity of compounds (7) and variability over time or in response to abiotic or biotic stimuli (8–10). Hence, it is often necessary to collect exometabolomic data across many replicates, environmental conditions, time points, and combinations of cell types or species. While high-throughput techniques for mass spectrometry currently exist (11), there remains a dearth of easily accessible methods for capturing exometabolomes in parallel across many conditions with limited sample manipulation (12). In particular, there are few approaches for scaling coculturing to thousands of combinations of organisms or cell types that interface with standard high-throughput instrumentation.

Growing different organisms together is often difficult because they compete for the same space and resources, resulting in a loss of diversity. Traditional coculturing experiments are carried out in liquid bioreactors or transwell systems (13), which may employ a membrane to physically separate different organisms or cell types. Cocultures can range in size from liters to microliters, allowing for considerable high-throughput replication of experiments. For example, the nanoporous microscale microbial incubator device consists of interconnected microwells that permit intercellular communication while maintaining physical separation (14). Although such systems avoid competition for space, they do not capture the exometabolome separately from the growing cells. Hence, these liquid systems typically necessitate laborious procedures to isolate, concentrate, purify, or extract the exometabolome (15). Coculturing on solid medium is sometimes preferable because spatial structuring is preserved and the exometabolome can be captured in a spatially explicit manner (16–18). Solid medium also elicits the production of natural products that many microorganisms will not readily produce in liquid media (19). A major drawback of culturing on solid media is that it is often low throughput, as methods relying on petri dishes fail to scale to the number of samples required to comprehensively study intercellular interactions among many cell types or species. Microscale platforms have been developed to miniaturize coculturing (20), but these systems require customization and machining that present barriers to their widespread adoption.

To address these challenges, we developed a technique called subtapping, short for substrate tapping, which is analogous to wiretapping (i.e., listening in on) but for a growth substrate. Subtapping uses a versatile three-dimensional (3D) printed platform, called SubTap, which we engineered to tap into the communal growth substrate shared by adjacent growth compartments. The SubTap platform is designed to adapt to experimental protocols that make use of a standard 96-well plate format. Since the SubTap design schematics are open source, it is easily modified to allow splitting of each well into one to four compartments for growing different organisms or cell types in mixed or unmixed cocultures. SubTap can be 3D printed at low cost, enabling the growth of thousands of cocultures with relatively little expense and effort in comparison to previous experimental systems. We demonstrate our approach’s ease of use by employing a mixture of known compounds at different concentrations. Coupling the SubTap with high-throughput mass spectrometry, we characterize cocultures of five different bacterial strains and putatively identify compounds attributable to different organisms. Taken together, these features empower SubTap users to coculture at previously intractable experimental scales and make way for the decrypting of complex microbial interactions.

## RESULTS

We sought to develop a relatively inexpensive technique for coculturing in high throughput and acquiring exometabolomic data by mass spectrometry. To this end, we modeled prototypes based on standard 96-well plates for their compatibility with existing protocols and equipment. Designs were developed in OpenSCAD and 3D printed on an Ultimaker 2+ with polylactic acid (PLA), which is automatically sterilized during the printing process (21). Importantly, PLA had no noticeable effect on bacterial growth compared to standard 96-well plates (see Fig. S1 in the supplemental material). To investigate further, we compared the final colony area after 64 h of growth for 12 replicates of the three tested strains that did not completely cover the well during this time and found no statistically significant difference (Mann-Whitney test *P* values of 0.82, 0.93, and 0.20).

10.1128/mSystems.00902-21.1 FIG S1 Movie strips of bacterial growth on traditional versus 3D printed 96-well plates with nutrient broth agar medium. Curves show the area of bright pixels in each well as a proxy for growth. Images are shown at regular intervals from 0 to 2.7 days of growth. Download FIG S1, PDF file, 0.5 MB. Copyright © 2021 Birer-Williams et al. 2021 Birer-Williams et al. https://creativecommons.org/licenses/by/4.0/ This content is distributed under the terms of the Creative Commons Attribution 4.0 International license.

**FIG 1:**
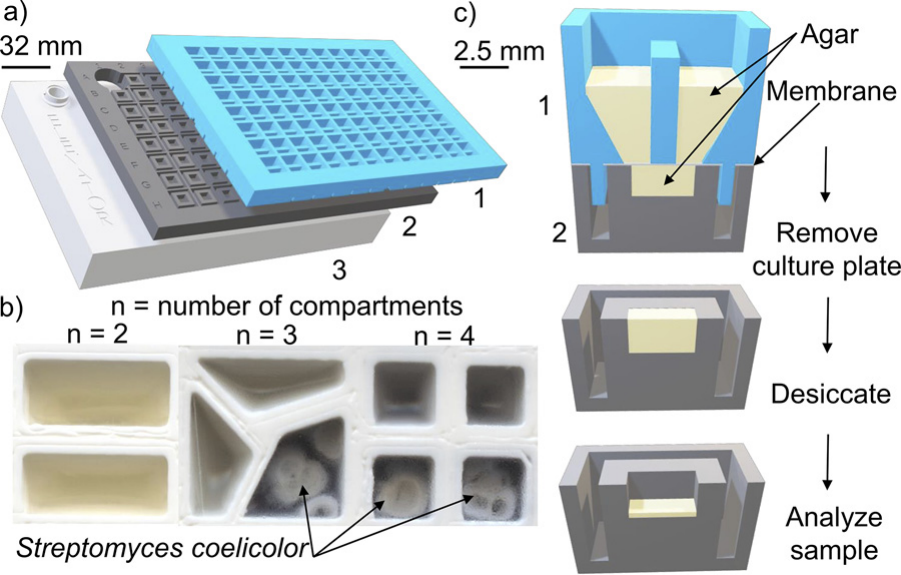
Views of the SubTap platform. (a) The culture plate (1) interlocks with an analysis plate (2) and an elevator plate (3). (b) Wells of the culture plate can be divided in two, three, or four compartments. The pigmented exometabolome of S. coelicolor diffuses through the soft agar and the 0.2-μm membrane, resulting in a color change in adjacent compartments absent bacteria. Separate wells are merged into a single image here for clarity. (c) A cross-sectional view of the platform. The well is divided into two compartments to separate the growth of different bacteria. The exometabolome of both bacteria diffuses through 60 μl of agar and the 0.2-μm membrane into the 20 μl of agar below. The exometabolome is captured after removal of the culture plate. The agar in the analysis plate is dried prior to analysis.

We iteratively modified the SubTap design after testing each prototype in the laboratory. The final SubTap design consisted of two interlocking plates (Fig. 1a), the top culture plate and the bottom analysis plate, separated by a 0.2-μm membrane. The membrane prevents bacteria that can grow into agar from traversing between compartments and enables us to easily disconnect the growth substrate from the cells. Each well of the culture plate is split into one to four compartments per user specification and filled with agar medium (Fig. 1b). Wells are separated by 2-mm-thick walls to prevent interwell leakage. A key design feature is that the membrane is printed within the culture plate by pausing the 3D printer during the print and gluing down a 0.2-μm membrane before printing resumes. This membrane is then cut along grooves between wells to avoid the possibility of cross-contamination due to wicking. Each SubTap was fabricated at a cost of $5.71 (6¢/sample) using a standard 3D printer, which includes $2.88 for 3D printer filament and $2.83 for membrane.

Well A1 was used to hold a vial of external standard that serves as a quality control (QC) for mass spectrometry. Thus, the analysis plate consists of 95 sample wells that each contain 30 μl of agar. Separate compartments on each well of the culture plate are inoculated with different strains or cell types. Cells communicate by secreting compounds that diffuse through the membrane, into the agar pad below, and between adjacent compartments, which permits intercellular communication without competition for space. As shown in Fig. 1c, the culture plate is removed after cell growth, and the exometabolome is captured in the 95 agar pads on the analysis plate below. These agar pads can then be rapidly dried and frozen before analysis. We chose to perform nanoelectrospray ionization (nESI) direct-infusion mass spectrometry (DIMS) for its relatively low cost and rapid analysis time (22), although the SubTap platform is compatible with other techniques capable of handling standard microwell plates. Briefly, we used liquid extraction surface analysis mass spectrometry (LESA-MS) to directly extract metabolites and inject the solvent into a mass spectrometer via nESI (23). Untargeted metabolomic data were acquired using a spectral stitching technique (24) for a total collection time of less than 5 min per sample (see Materials and Methods). We chose to use MS1 as a high-throughput approach for screening purposes, which tolerate a higher false discovery rate, and follow-up with lower-throughput techniques for validation.

### Detection of a known compound mixture by subtapping.

**FIG 2:**
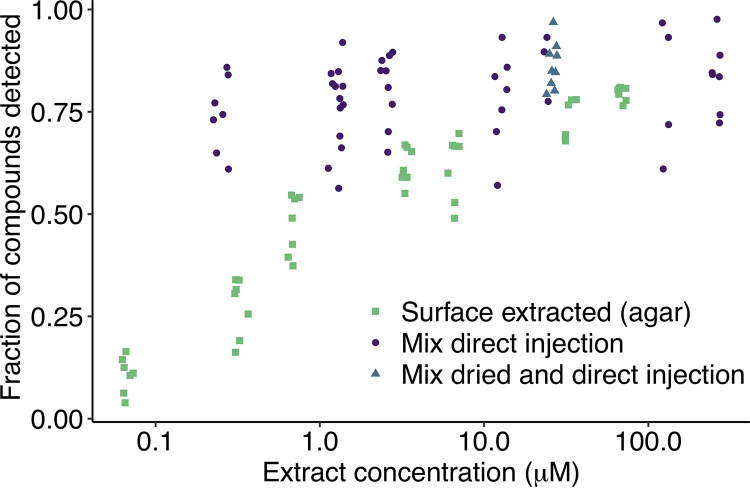
The proportion of known compounds detected decreased with compound concentration. Mixtures of 26 known compounds after 2 days of diffusion through agar (green squares) in the SubTap platform became less detectable at lower concentration. In contrast, direct injection of the compound mixture (purple circles) still allowed for a high fraction of the compounds to be detected at lower concentrations. Drying the compound mixture (blue diamonds) prior to direct injection had no discernible effect. Note the log scaled *x* axis. Random jitter was added to points in the *x* direction for clarity.

We characterized SubTap using a mixture of 26 known compounds across seven concentrations (Fig. 2). Almost all compounds were putatively detected in at least one of the seven replicates (1 to 6 adducts per compound; 5 ppm tolerance; see Table S1 in the supplemental material). Four compounds were not detected at any concentration within the surface-extracted agar samples, but they were putatively detected by direct mixture injection (Table S2 and Fig. S2). We hypothesized that these compounds were undetectable due to charge competition with molecules coextracted from the agar (25), indicating that some exometabolites might be challenging to detect in the presence of an agar background. At the highest concentration (66.7 μM) of compounds tested using the SubTap platform, an average of 80% ± 2% of the 26 compounds were putatively detected per replicate, which was comparable to directly injecting the mixture of 26 compounds without use of the platform at 125 μM or 25 μM (81% ± 16% and 86% ± 8%, respectively). However, at lower concentrations (≤1 μM), fewer compounds were detected using SubTap (47% ± 7% at 0.67 μM) than through direct injection of the 26-compound mixture (74% ± 9% at 0.25 μM). We tested whether this effect was due to the drying step associated with the SubTap process, but drying the 26-compound mixture, resolubilizing in extraction solvent, and then injecting at 25 μM did not cause any loss of detection compared to directly injecting the mixture (86% ± 6% and 86% ± 8%, respectively). We suspect that ion suppression from compounds in the agar is the primary reason for decreased detection at lower concentrations relative to the direct injection method. Notwithstanding this limitation, our approach permitted the detection of approximately half of the compounds at 1 μM concentration.

10.1128/mSystems.00902-21.2 FIG S2 Heatmap of the percent of compounds detected among replicates for the three tests: surface extracted (agar), mix direct injection, and dried direct injection. Download FIG S2, PDF file, 0.2 MB. Copyright © 2021 Birer-Williams et al. 2021 Birer-Williams et al. https://creativecommons.org/licenses/by/4.0/ This content is distributed under the terms of the Creative Commons Attribution 4.0 International license.

10.1128/mSystems.00902-21.7 TABLE S1 Table of the 23 adduct possibilities searched for the 26 known compounds. Download Table S1, PDF file, 0.03 MB. Copyright © 2021 Birer-Williams et al. 2021 Birer-Williams et al. https://creativecommons.org/licenses/by/4.0/ This content is distributed under the terms of the Creative Commons Attribution 4.0 International license.

10.1128/mSystems.00902-21.8 TABLE S2 The mean number of adducts detected for each compound among mixtures with seven replicates. Download Table S2, PDF file, 0.06 MB. Copyright © 2021 Birer-Williams et al. 2021 Birer-Williams et al. https://creativecommons.org/licenses/by/4.0/ This content is distributed under the terms of the Creative Commons Attribution 4.0 International license.

### High repeatability of coculture exometabolomes by subtapping.

**FIG 3:**
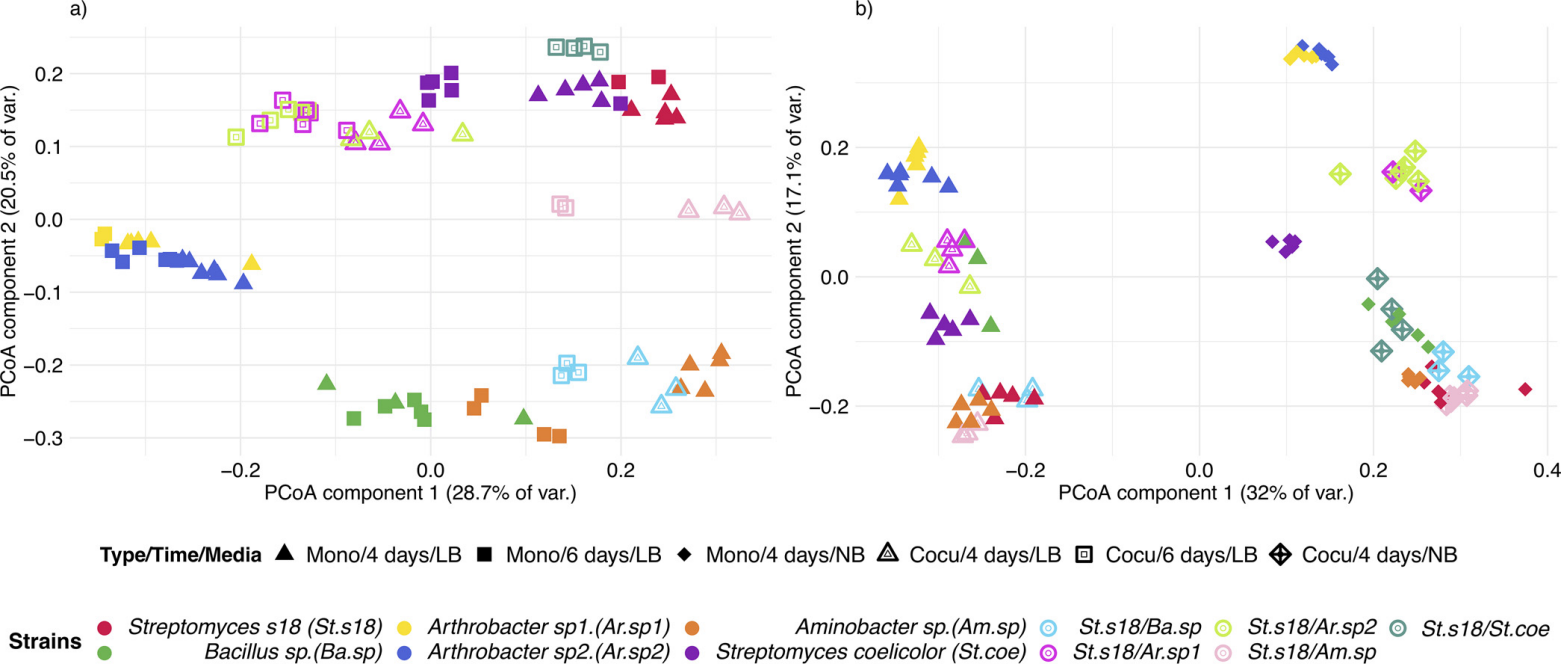
Clustering of samples into groups of biological replicates. Principal coordinate analysis (PCoA) of the matrix of Bray-Curtis sample dissimilarities captures differences in the diversity of the exometabolome. The tight clustering of biological replicates validates SubTap's utility for exometabolomics. (a) The exometabolome of six soil bacteria grown on LB medium varied with the duration of growth, 4 or 6 days, and strain interactions. (b) The exometabolome of six soil bacteria at 4 days on LB and NB media varied with growth medium and strain interactions. var., variance; Mono, monoculture; Cocu, coculture.

Having characterized SubTap’s performance with known compounds, we next sought to validate the platform with biological samples. To this end, we employed a panel of six soil bacteria, including two sets of two bacteria that each belong to the same genus, as diverse representatives of the microbiome (Fig. S3). Strains were grown alone and in pairwise competition via adjacent compartments connected through the same analysis plate well. Exometabolomes were captured on two different media (Luria-Bertani [LB] and nutrient broth [NB]) and on LB at two different time points (4 and 6 days after inoculation), with wells divided in two compartments for unmixed culture of two strains or with the same strain in each compartment for monoculture. As expected, principal coordinate analysis (PCoA) of the exometabolomes revealed a clustering of samples into groups of biological replicates that were more like each other than other samples (Fig. 3).

10.1128/mSystems.00902-21.3 FIG S3 Maximum likelihood phylogenetic tree built from the 16S ribosomal RNA gene of the six bacterial strains used in this study. Download FIG S3, PDF file, 0.08 MB. Copyright © 2021 Birer-Williams et al. 2021 Birer-Williams et al. https://creativecommons.org/licenses/by/4.0/ This content is distributed under the terms of the Creative Commons Attribution 4.0 International license.

We performed permutational multivariate analysis of variance (PERMANOVA) to analyze the effect of experimental variables on the clustering of exometabolomes. In the experiment comparing time points (Fig. 3a), the strain explained 74.3% of the variance, the time of collection explained 5.7%, and the interaction of time and strain explained 7.5%, leaving 12.5% of the variance unexplained (time, *F*1,86 = 29.92, *P* < 0.001, *R*^2^ = 0.057; strains, *F*10,86 = 39.22, *P* < 0.001, *R*^2^ = 0.743; interaction of time and strains, *F*9,86 = 4.42, *P* < 0.001, *R*^2^ = 0.075). We believe the relatively small effect of collection time on the exometabolome could be due to the exometabolome reaching steady state before the first time point or the disproportionate influence of strain compared to sampling time. In the experiment comparing LB and NB media (Fig. 3b), the type of medium explained 30.9% of the variance in *m*/*z* features, the strain explained another 43.8%, and the interaction of media and strains explained 18.9%, leaving only 6.4% of the variance unexplained (media, *F*1,96 = 368.88, *P* < 0.001, *R*^2^ = 0.309; strains, *F*10,96 = 52.33, *P* < 0.001, *R*^2^ = 0.437; interaction of media and strains, *F*9,96 = 25.19, *P* < 0.001, *R*^22^ = 0.189). These results validated SubTap’s utility for coculturing and our ability to extract meaningful information due to the high repeatability of exometabolomes across biological replicates.

### Putative identification of exometabolites with tandem MS.

Given that groups of cultures were distinguishable by their mass spectra (MS1), we wished to identify *m*/*z* features only present in specific culture groups. To this end, we selected the most intense *m*/*z* features detected only in one or two monoculture or coculture samples from the 11 sample groups belonging to the experiment on NB medium after 4 days of growth (e.g., Fig. S4). We performed tandem mass spectrometry (MS2) using the two remaining replicates (of eight total) belonging to each of the 11 coculture groups, resulting in successful acquisition of MS2 data for 80% to 90% of targeted parent (MS1) ions per sample. These spectra were submitted to the GNPS platform (26), which putatively identified 62 of the *m*/*z* features (Table S3). We focused on four compounds of relatively high molecular weight because our targeted MS1 spectra in direct infusion were devoid of interfering peaks in these regions. Two putative identifications were the siderophores proferrioxamine G1t (*m*/*z* 520.3554) and desferrioxamine E (*m*/*z* 588.2620), which were found only in cultures containing Streptomyces coelicolor. Similarly, the siderophore desferrioxamine B (*m*/*z* 585.8080) was found only in cultures containing *Arthrobacter* species 2 (sp2), and coelichelin (*m*/*z* 601.2949) in cultures containing *Bacillus* sp.

10.1128/mSystems.00902-21.4 FIG S4 Intensity (MS1 data set) of the four *m*/*z* features with putative identifications (in the GNPS database) across the 11 biological groups. The four siderophores were proferrioxamine G1t (*m*/*z* 520.3554), desferrioxamine B (*m*/*z* 585.8080), desferrioxamine E (*m*/*z* 588.2620), and coelichelin (*m*/*z* 601.2949). Download FIG S4, PDF file, 0.1 MB. Copyright © 2021 Birer-Williams et al. 2021 Birer-Williams et al. https://creativecommons.org/licenses/by/4.0/ This content is distributed under the terms of the Creative Commons Attribution 4.0 International license.

10.1128/mSystems.00902-21.9 TABLE S3 Putative identifications of 62 *m*/*z* features using GNPS on samples from NB medium after 4 days of growth. Download Table S3, PDF file, 0.07 MB. Copyright © 2021 Birer-Williams et al. 2021 Birer-Williams et al. https://creativecommons.org/licenses/by/4.0/ This content is distributed under the terms of the Creative Commons Attribution 4.0 International license.

### Mixed versus unmixed cocultures have distinct exometabolomes.

A major advantage of SubTap’s versatility is that strains can be easily grown in mixed (one compartment) or unmixed (two or more compartments) coculture. Unmixed coculturing permits the strains to grow without competition for space, which assists with coculturing when there are large differences in growth rates or lag times. PCoA of exometabolomes revealed substantial differences between mixed and unmixed cocultures (Fig. S5a). *Bacillus* dominated each of its mixed cocultures, which exhibited similar exometabolomes to the *Bacillus* monoculture. In contrast, the exometabolomes of unmixed cocultures with *Bacillus* and other strains never clustered with the *Bacillus* monoculture (adjusted *P* value [p-adj] < 0.004; Tukey’s test), suggesting *Bacillus* could not dominate without competition for space. Similarly, mixed cocultures between Streptomyces coelicolor and *Arthrobacter* sp2 or *Streptomyces* strain 18 (s18) clustered with the S. coelicolor monoculture, but unmixed cultures did not (p-adj < 0.003).

10.1128/mSystems.00902-21.5 FIG S5 Mixed and unmixed culturing with SubTap. (a) When mixed with *Streptomyces* s18 (*S.* s18) and/or *Arthrobacter* in the same compartment, S. coelicolor and *Bacillus* clearly dominated the exometabolome, but when S. coelicolor or *Bacillus* where mixed, the exometabolome appeared between those of either strain's monoculture. In contrast, unmixed culturing resulted in exometabolomes that did not cluster with the monoculture of any individual strain. (b) Cells on the SubTap can be cocultured in up to four separate compartments. Both S. coelicolor and *Bacillus* dominated unmixed cultures unless they were grown together, in which case *Bacillus* dominated. Download FIG S5, PDF file, 0.3 MB. Copyright © 2021 Birer-Williams et al. 2021 Birer-Williams et al. https://creativecommons.org/licenses/by/4.0/ This content is distributed under the terms of the Creative Commons Attribution 4.0 International license.

The customizability of SubTap enables wells of the culture plate to be divided into three or four equal-area compartments for unmixed multiculturing. We used PCoA to compare the exometabolomes of every possible combination of four strains (Fig. S5b). S. coelicolor and *Bacillus* clearly dominated the exometabolomes when they were grown with *Streptomyces* s18 and/or *Arthrobacter*. However, whenever S. coelicolor or *Bacillus* was grown together (unmixed), the exometabolome appeared between those of either strain individually (Fig. S5b). Taken together, these results highlight the merits of unmixed subtapping for studying coculture exometabolomes in cases where competition for space may prevent mixed growth.

To further investigate this data set, we identified the subset of 43 observed *m*/*z* features (Table S4) matching compounds that are known to be produced by S. coelicolor present in StreptomeDB (27). Several of these *m*/*z* features were observed only in samples containing S. coelicolor (Fig. S6). For example, we observed the antibiotic actinorhodin along with its precursors {e.g., 4-dihydro-9-hydroxy-1-methyl-10-oxo-3-H-naptho-[2,3-c]-pyran-3-(*S*)-acetic acid [(*S*)-DNPA] and kalafungin (28)}. The precursors were always present with S. coelicolor and absent without, except when S. coelicolor was mixed with *Bacillus* in pairwise coculture where *Bacillus* dominated. In contrast, the actinorhodin final product was observed only when S. coelicolor was grown with two or three other strains, suggesting its production might be elicited by increased competition.

10.1128/mSystems.00902-21.6 FIG S6 Putatively identified *m*/*z* features associated with Streptomyces coelicolor samples. Observed *m*/*z* features matching those produced by S. coelicolor (present in StreptomeDB) are shown by observed intensity across samples. Some of the compounds are not observed in the absence of S. coelicolor or in mixed coculture with *Bacillus* where it is believed S. coelicolor was outcompeted. Download FIG S6, PDF file, 0.5 MB. Copyright © 2021 Birer-Williams et al. 2021 Birer-Williams et al. https://creativecommons.org/licenses/by/4.0/ This content is distributed under the terms of the Creative Commons Attribution 4.0 International license.

10.1128/mSystems.00902-21.10 TABLE S4 Putative identifications of 43 *m*/*z* features in Streptomyces coelicolor cultures using StreptomeDB. Download Table S4, PDF file, 0.04 MB. Copyright © 2021 Birer-Williams et al. 2021 Birer-Williams et al. https://creativecommons.org/licenses/by/4.0/ This content is distributed under the terms of the Creative Commons Attribution 4.0 International license.

### Attributing production of *m*/*z* features to specific strains.

**FIG 4:**
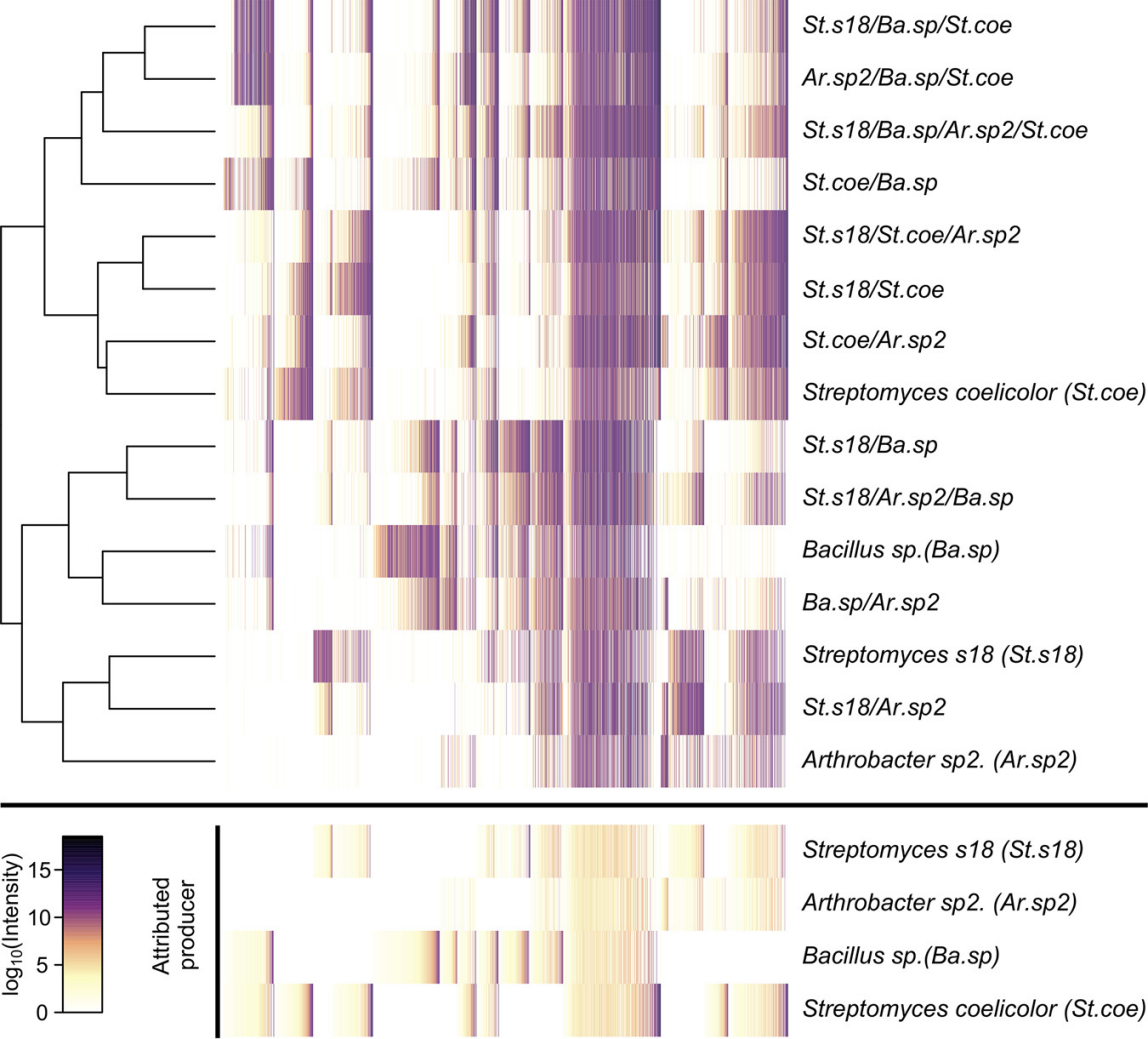
Attribution of *m*/*z* features to specific strains. The average intensity of each *m*/*z* feature is shown for the (unmixed) coculturing of four strains on NB medium after 6 days of growth. Samples (rows) are clustered based on their similarity, as shown by the dendrogram. This matrix of intensities was used in conjunction with the known presence or absence of each organism to solve for the amount of intensity attributable to each strain (bottom). Each column represents an *m*/*z* feature, with (top and bottom) columns sorted by their attributed producers and intensity (bottom). Many *m*/*z* features were produced by all strains, while a subset was attributable to a single strain.

We considered whether it would be feasible to capitalize on SubTap’s design to pinpoint which strain was responsible for the production of each *m*/*z* feature. We formulated this problem as solving for each strain’s contribution to every *m*/*z* feature given the known presence or absence of strains across samples (see Materials and Methods). This required the assumption that all strains grew when present, so we limited the analysis to unmixed cocultures where strains were grown in separate, but connected, compartments. This resulted in a matrix of attributable intensities for each *m*/*z* feature, with many features being attributable to more than one strain (Fig. 4). In total, 17% of *m*/*z* features were attributable to all four strains, 24% to three strains, 36% to two strains, and 23% were attributable to a single strain. S. coelicolor was attributed to 63% of *m*/*z* features, *Bacillus* to 60%, *Streptomyces* s18 to 60%, and *Arthrobacter* to 52%. Overall, this analysis demonstrated the ability to attribute the contribution of specific *m*/*z* features to individual strains using coculture mass spectrometry data.

## DISCUSSION

The SubTap platform fills a void in high-throughput coculturing by enabling flexible experimental designs with many replicates for relatively low cost. In principle, subtapping could be used with other analytical techniques to capture different facets of intercellular communication. We chose to use DIMS for higher throughput, albeit lower molecular coverage, than other techniques utilizing pre-mass analysis chromatographic separations. The collection window (±1 *m*/*z*) of parent ions made it difficult to capture clean MS2 spectra with direct injection, because it required high intensity targets without nearby competitor peaks of similar intensity. Nevertheless, SubTap is designed to work with most experimental instruments that can accommodate standard 96-well plate format, and pre-mass analysis chromatography steps can readily be adapted to minimize MS1 overlap and increase confidence in molecular annotations of detected ions. These chromatography steps are not limited to conventional methods like liquid or gas chromatography. The SubTap works readily with commercially available automated solid-phase extraction devices that can be coupled to mass spectrometers (29), even those with postionization/pre-mass analysis ion mobility separation (30).

The use of 3D printing permits customizable experimental designs. Source files for SubTap are available online (https://www.thingiverse.com/thing:4644466), and users only need to specify the number of compartments required in each of the sample wells. The downside of 3D printing is its relatively slow speed, requiring a little less than 1 day to print each SubTap on a standard 3D printer. Injection molding would present a significantly faster solution for large-scale production. Still, SubTap in many ways represents an advance over previous coculturing designs that require machining, nonstandard equipment, or significant labor. Production of the SubTap is relatively labor free, which greatly reduces its total cost relative to many other coculturing devices and is accessible to many research laboratories thanks to the low cost of 3D printers. We anticipate that SubTap can be widely adopted for many purposes and unlock new discoveries in intercellular communication.

## MATERIALS AND METHODS

### SubTap, a 3D printed interlocked platform to capture the exometabolome.

We have developed and characterized SubTap, a 3D printed platform for capturing the exometabolome of microorganisms in high throughput. SubTap was designed using OpenSCAD (v2015.03) and then transformed with Cura (v3.5) for printing with polylactic acid (PLA; MatterHackers) 3-mm filament using an Ultimaker 2+ 3D printer. We printed all pieces using a 0.4-mm nozzle and 50% infill. As shown in Fig. 1a, the platform is composed of two 3D printed plates that interlock. The culture plate (Fig. 1a) has 96 square wells that can be divided into two, three, or four compartments by a 2-mm wall (Fig. 1b), allowing the unmixed coculture of two or more different bacterial strains in each well. The culture plate contains 60 μl of soft (0.5%) agar medium per compartment when each well is divided into two or three compartments and 40 μl when divided into four compartments. Upon assembly, the culture plate is manually clipped into the analysis plate (Fig. 1a), which contains 95 square wells and a space in well A1 for a typical high performance liquid chromatography (HPLC) autosampler vial containing an external defined quality control. The analysis plate contains 30 μl of soft agar per well. During 3D printing, a 0.2-μm polycarbonate track-etched membrane filter (GVS; Sanford) is embedded in the culture plate by pausing at a layer height of 7.2 mm and gluing down the membrane before resuming the print. This membrane allows for the diffusion of metabolites into the analysis plate, while preventing microorganisms from growing into the bottom wells. The bottom of the culture plate contains grooves that assist in quickly cutting the membrane between wells to avoid cross-contamination through wicking. In order to adjust the elevation of the analysis plate for automated sampling, the analysis plate is placed on top of an elevator plate (Fig. 1a), which can be reused for many analysis plates.

### Mixture of 26 known compounds.

To assess technical variability and characterize the platform, we tested our ability to detect 26 known compounds in equimolar mixes at different concentrations. The 26 known compounds selected for this study range from an exact mass of 121.0738 to 733.4612 g/mol (see Table S2 in the supplemental material). Equimolar solutions containing all compounds were created at seven different concentrations: 1,000, 500, 100, 50, 10, 5, and 1 μM. We added 5 μl of each mix in both compartments (10 μl per well), with seven technical replicates for each mixture concentration. The SubTap was then covered by a thin layer of parafilm to prevent desiccation and incubated for 2 days at 37°C to mimic conditions of microbial metabolite diffusion in the soft agar. After removal of the culture plate, agar pads on the analysis plate were dried under a fan at room temperature for 2 h and then stored at −20°C. Samples were automatically extracted with 15 μl of 70% methanol (MeOH) and 0.1% formic acid using an Advion TriVersa Nanomate and injected into a Thermo Velos mass spectrometer as described below.

Under the assumption that the diffusion of all compounds was homogenous in the agar, and the extraction is 100% efficient, a maximum of 66.67, 33.33, 6.67, 3.33, 0.67, 0.33, and 0.07 μM concentration solutions were directly injected. To test whether the undetected compounds were a consequence of experimental processes (i.e., diffusion, extraction, and matrix effects) or of ionization issues, 10-μl portions of seven replicates of each mix were diluted to 25% with 70% MeOH and 0.1% formic acid and directly injected into the mass spectrometer. This resulted in 250, 125, 25, 12.5, 2.5, 1.25, and 0.25 μM solutions directly injected in the mass spectrometer. To test whether the undetected compounds resulted from desiccation of the agar pad, five replicates of the mix at a concentration of 100 μM were diluted to 25% with 70% MeOH before drying and resuspending in the same volume of 70% MeOH, resulting in a 25 μM solution directly injected in the mass spectrometer.

All statistical analyses were carried out in R (v4.0.2) (31). In order to detect how many of the 26 known compounds had diffused through the system, we searched for 23 possible adducts (Table S1) with a tolerance of 5 ppm using the function *match.closest* in the MALDIquant package (32). A compound was considered detected if one or more adducts were identified in a well.

### Coculturing of soil isolates.

The SubTap can be used to culture cells in a controlled environment with stimuli of interest (e.g., drug candidates, antibiotics, extracts). We isolated five bacteria from the same soil sample and identified them as *Streptomyces* s18 (St. s18), *Bacillus* sp. (Ba. sp), *Arthrobacter* sp1 (Ar. sp1), *Arthrobacter* sp2 (Ar. sp2), and *Aminobacter* sp. (Am. sp) by sequencing their 16S rRNA gene (GenBank accession numbers MW041646 to MW041650). Streptomyces coelicolor M145 (St. coe) was used as a positive control. MS1 data were collected for six replicates of monocultures of each strain as well as pairwise cocultures of strain St. s18 with Ba. sp, Ar. sp1, Ar. sp2, Am. sp, and St. coe. To explore the ability of the platform to detect small changes in the exometabolome at different time points, two replicate SubTap plates containing Luria-Bertani (25 g/liter) agar medium were incubated for 4 days and 6 days. Similarly, a third SubTap was made with nutrient broth (8 g/liter) agar and incubated 4 days to evaluate the impact of medium on the exometabolome. For each sample, 5 μl of cell suspension containing 10^6^ cells per ml was added to both compartments of a well for monoculture, in separate compartments of a well for unmixed coculture, and 2.5 μl per strain mixed in each compartment of a well for mixed coculture. The SubTap was protected using parafilm or Breathe Easy membranes (Research Products International) to prevent desiccation before incubating at 28°C. Once the incubation period ended, the culture plate was removed, and agar pads on the analysis plate were dried under a fan at room temperature for 2 h before storing at −20°C.

To explore the utility of SubTap for use with more than two interacting strains in mixed or unmixed coculture, a fourth plate was tested with NB medium after 6 days of incubation. Here, four bacterial strains were selected for coculturing (St. s18, Ba. sp, Ar. sp2, and St. coe). This SubTap included monocultures of each strain, mixed cocultures of the four strains in pairs, and unmixed cultures of two to four strains. All other steps were identical to those previously described.

### Acquisition of direct-infusion mass spectrometry (DIMS) data.

All mass spectrometry was performed at the Environmental Molecular Science Laboratory on the campus of Pacific Northwest National Laboratory (PNNL). We used a Triversa Nanomate (Advion) coupled to a high-resolution MS (Thermo Scientific Velos LTQ Pro Orbitrap) to perform LESA-MS analysis (23). For all analyses, the 96-well plates were maintained at 10°C. The agar pads of the analysis plate were subjected to individual extraction by an organic solvent mixture containing 70% methanol and 0.1% formic acid. The pipette was loaded with 20 μl of the extraction solution, 15 μl was dispensed onto the agar pad, and the solution remained on the sample for 4 s before aspirating 5 μl back into the pipette. The extract was directly injected into the MS through the Advion nanochip (5-μm nominal internal diameter nozzle chip). In order to detect problems with injection or acquisition, an external defined quality control (part number G1969-85000; Agilent), stored in position A1, was injected every 10 samples.

MS data were acquired following the spectral-stitching method where data are measured in several overlapping *m*/*z* windows and subsequently “stitched” together to create a complete mass spectrum (24). Full mass spectra were taken across the entire mass range and followed by a series of 100 *m*/*z* wide window scans. A 30-s wait period was established to allow for nESI spray stabilization prior to acquisition. The mass spectrometer was configured with the following parameters: mass resolution at 200 *m*/*z* of 100 K, full scans of 70 to 1,200 *m*/*z*, and 16 total window scans of 100 *m*/*z* selected ion monitoring (SIM) with 30 *m*/*z* overlap between windows, all with a 10^6^ AGC target. Window scans across the *m*/*z* range required ∼15-s acquisition time. This process was repeated seven times per *m*/*z* window, the total time per injection was approximately 5 min.

### Processing of mass spectrometry data.

Mass spectra were processed by scan stitching, noise removal, peak picking, and peak alignment using the Python (v2.7.15) package DIMSpy (v1.3.0) and its standard operating procedure (24). First, SIM windows from 780 *m*/*z* to 1,200 *m*/*z* were discarded due to consistently low intensity. Then, the remaining SIM windows were stitched together, noise was removed with the *noise_packets* function using a minimum signal-to-noise ratio (SNR) of 3, and peaks were detected with a maximum mass tolerance of 3 ppm across replicate scans of the same sample. Finally, peaks were aligned across all samples with a maximum mass tolerance of 3 ppm in order to obtain a matrix of peak intensity by sample.

Sample injection control and filtering steps were carried out in R (v 4.0.2) (31). For sample injection control, principal coordinate analysis was performed on the pairwise Bray–Curtis dissimilarities of the peak matrix normalized by the sum of sample intensities. Defined QC samples were removed from the peak matrix after verifying that they clustered together and apart from other samples on the PCoA plot. Then, values were log transformed and filtered following two steps: filtering features present in blank (medium only) samples and filtering features based on the percentage of missing values. Background subtraction followed the data-adaptative procedure developed by Schiffman et al. (33) that considers the number of blanks in which each feature is detected and assigns cutoffs according to the background noise. Filtering based on blank samples was performed separately for each experiment. The *m*/*z* features which had more than 80% missing values throughout all samples were removed if they were also missing in more than 20% of biological replicates.

The effects of experimental factors and their interactions were assessed with a permutational multivariate analysis of variance (PERMANOVA) and visualized with principal coordinate analysis performed on the pairwise Bray-Curtis dissimilarities of the peak matrix. For some samples, multilevel pairwise comparison of PERMANOVA were run with false discovery rate correction on the pairwise Bray-Curtis dissimilarities of the peak matrix. Also, *m*/*z* features [M+H]^+^ found in samples with Streptomyces coelicolor were compared with the expected exact mass of [M+H]^+^ adducts of known compounds of S. coelicolor present in StrepomeDB (v3) (27) within 15 ppm.

We formulated the problem of attributing each *m*/*z* feature to its producer(s) as Ax=b, where A is the binary matrix of samples by bacteria (where 1 is present and 0 is absent), b is the log of average intensity for each *m*/*z* feature across replicate samples, and x is the amount of that intensity attributable to each bacterium. This formulation assumes the following. (i) The features are independent. (ii) All strains must have grown when present. (iii) If a strain can produce a feature, then it does. (iv) Variability in intensity is normally distributed in log space. If we assume *m*/*z* features cannot be destroyed (i.e., no negative production), then this problem can be solved using nonnegative least squares. We used the sequential coordinate-wise algorithm (34) to solve for x separately for each feature. The result was used to reorder a heatmap of the observed intensities such that *m*/*z* features were grouped by the strain(s) to which they were attributed (Fig. 4).

### Acquisition and processing of tandem mass spectrometry data.

Tandem mass spectrometry data were acquired for the experiment with NB medium at 4 days of growth. We selected *m*/*z* features present at high intensity in 100% of the six replicates of one or two samples and absent in all other samples (e.g., see Fig. S5 in the supplemental material). This resulted in 11 lists of targeted *m*/*z* features for tandem mass spectrometry on the two remaining sample replicates of each monoculture and coculture. Extraction and injection in the mass spectrometer were identical to the procedure for acquiring MS1 data (see above), but the MS data acquisition method was modified to acquire MS2 between full spectral scans. Specifically, an overall MS scan was collected and then another every seventh scan thereafter. For the interim six MS scans, collision-induced dissociation (CID) fragmentation was performed on the target peaks if it was detected in the full spectral scan (using a new target peak every cycle). The acquisition time per injection was 12 min.

Tandem mass spectrometry data were processed under R with package xcms (35), after files were transformed into mzml format with ProteoWizard (v3) (36). To control the percentage of targeted *m*/*z* features leading to the acquisition of fragmentation spectra, mzml files were processed with the function *match.closest* from MALDIquant (32). Also, mzml files were uploaded to GNPS to run a spectral library search leading to the putative identification of compounds (26). Library matches were kept if their cosine score was at least 0.7 and they shared six or more fragments.

## References

[1] Silva LP, Northen TR. 2015. Exometabolomics and MSI: deconstructing how cells interact to transform their small molecule environment. Curr Opin Biotechnol 34:209–216. doi:10.1016/j.copbio.2015.03.015. 25855407

[2] Swenson TL, Karaoz U, Swenson JM, Bowen BP, Northen TR. 2018. Linking soil biology and chemistry in biological soil crust using isolate exometabolomics. Nat Commun 9:19. doi:10.1038/s41467-017-02356-9. 29296020 PMC5750228

[3] Medlock GL, Carey MA, McDuffie DG, Mundy MB, Giallourou N, Swann JR, Kolling GL, Papin JA. 2018. Inferring metabolic mechanisms of interaction within a defined gut microbiota. Cell Syst 7:245–257.e247. doi:10.1016/j.cels.2018.08.003. 30195437 PMC6166237

[4] Alsufyani T, Weiss A, Wichard T. 2017. Time course exo-metabolomic profiling in the green marine macroalga Ulva (Chlorophyta) for identification of growth phase-dependent biomarkers. Mar Drugs 15:14. doi:10.3390/md15010014. PMC5295234 28075408

[5] Fu ZB, Verderame TD, Leighton JM, Sampey BP, Appelbaum ER, Patel PS, Aon JC. 2014. Exometabolome analysis reveals hypoxia at the up-scaling of a Saccharomyces cerevisiae high-cell density fed-batch biopharmaceutical process. Microb Cell Fact 13:32. doi:10.1186/1475-2859-13-32. 24593159 PMC4016033

[6] Sue T, Obolonkin V, Griffiths H, Villas-Bôas SG. 2011. Villas-Boas SG: an exometabolomics approach to monitoring microbial contamination in microalgal fermentation processes by using metabolic footprint analysis. Appl Environ Microbiol 77:7605–7610. doi:10.1128/AEM.00469-11. 21890679 PMC3209156

[7] Costa CP, Goncalves Silva D, Rudnitskaya A, Almeida A, Rocha SM. 2016. Shedding light on Aspergillus niger volatile exometabolome. Sci Rep 6:27441. doi:10.1038/srep27441. 27264696 PMC4893740

[8] Marasco CC, Enders JR, Seale KT, McLean JA, Wikswo JP. 2015. Real-time cellular exometabolome analysis with a microfluidic-mass spectrometry platform. PLoS One 10:e0117685. doi:10.1371/journal.pone.0117685. 25723555 PMC4344306

[9] Senges CHR, Al-Dilaimi A, Marchbank DH, Wibberg D, Winkler A, Haltli B, Nowrousian M, Kalinowski J, Kerr RG, Bandow JE. 2018. The secreted metabolome of Streptomyces chartreusis and implications for bacterial chemistry. Proc Natl Acad Sci USA 115:2490–2495. doi:10.1073/pnas.1715713115. 29463727 PMC5877972

[10] Traxler MF, Watrous JD, Alexandrov T, Dorrestein PC, Kolter R. 2013. Interspecies interactions stimulate diversification of the Streptomyces coelicolor secreted metabolome. mBio 4:e00459-13. doi:10.1128/mBio.00459-13. 23963177 PMC3747584

[11] de Raad M, Fischer CR, Northen TR. 2016. High-throughput platforms for metabolomics. Curr Opin Chem Biol 30:7–13. doi:10.1016/j.cbpa.2015.10.012. 26544850

[12] Goers L, Freemont P, Polizzi KM. 2014. Co-culture systems and technologies: taking synthetic biology to the next level. J R Soc Interface 11:20140065. doi:10.1098/rsif.2014.0065. 24829281 PMC4032528

[13] Adnani N, Chevrette MG, Adibhatla SN, Zhang F, Yu Q, Braun DR, Nelson J, Simpkins SW, McDonald BR, Myers CL, Piotrowski JS, Thompson CJ, Currie CR, Li L, Rajski SR, Bugni TS. 2017. Coculture of marine invertebrate-associated bacteria and interdisciplinary technologies enable biosynthesis and discovery of a new antibiotic, keyicin. ACS Chem Biol 12:3093–3102. doi:10.1021/acschembio.7b00688. 29121465 PMC5973552

[14] Ge Z, Girguis PR, Buie CR. 2016. Nanoporous microscale microbial incubators. Lab Chip 16:480–488. doi:10.1039/c5lc00978b. 26584739

[15] Kosina SM, Danielewicz MA, Mohammed M, Ray J, Suh Y, Yilmaz S, Singh AK, Arkin AP, Deutschbauer AM, Northen TR. 2016. Exometabolomics assisted design and validation of synthetic obligate mutualism. ACS Synth Biol 5:569–576. doi:10.1021/acssynbio.5b00236. 26885935

[16] Moree WJ, Phelan VV, Wu CH, Bandeira N, Cornett DS, Duggan BM, Dorrestein PC. 2012. Interkingdom metabolic transformations captured by microbial imaging mass spectrometry. Proc Natl Acad Sci USA 109:13811–13816. doi:10.1073/pnas.1206855109. 22869730 PMC3427086

[17] Yang JY, Phelan VV, Simkovsky R, Watrous JD, Trial RM, Fleming TC, Wenter R, Moore BS, Golden SS, Pogliano K, Dorrestein PC. 2012. Primer on agar-based microbial imaging mass spectrometry. J Bacteriol 194:6023–6028. doi:10.1128/JB.00823-12. 22821974 PMC3486372

[18] Sica VP, Raja HA, El-Elimat T, Kertesz V, Van Berkel GJ, Pearce CJ, Oberlies NH. 2015. Dereplicating and spatial mapping of secondary metabolites from fungal cultures in situ. J Nat Prod 78:1926–1936. doi:10.1021/acs.jnatprod.5b00268. 26192135 PMC4570219

[19] Edgar RC. 2018. Updating the 97% identity threshold for 16S ribosomal RNA OTUs. Bioinformatics 34:2371–2375. doi:10.1093/bioinformatics/bty113. 29506021

[20] Barkal LJ, Theberge AB, Guo C-J, Spraker J, Rappert L, Berthier J, Brakke KA, Wang CCC, Beebe DJ, Keller NP, Berthier E. 2016. Microbial metabolomics in open microscale platforms. Nat Commun 7:10610. doi:10.1038/ncomms10610. 26842393 PMC4742997

[21] Neches RY, Flynn KJ, Zaman L, Tung E, Pudlo N. 2016. On the intrinsic sterility of 3D printing. PeerJ 4:e2661. doi:10.7717/peerj.2661. 27920950 PMC5136128

[22] Ren J-L, Zhang A-H, Kong L, Wang X-J. 2018. Advances in mass spectrometry-based metabolomics for investigation of metabolites. RSC Adv 8:22335–22350. doi:10.1039/C8RA01574K. PMC9081429 35539746

[23] Velickovic D, Chu RK, Carrell AA, Thomas M, Pasa-Tolic L, Weston DJ, Anderton CR. 2018. Multimodal MSI in conjunction with broad coverage spatially resolved MS(2) increases confidence in both molecular identification and localization. Anal Chem 90:702–707. doi:10.1021/acs.analchem.7b04319. 29210566

[24] Southam AD, Weber RJM, Engel J, Jones MR, Viant MR. 2016. A complete workflow for high-resolution spectral-stitching nanoelectrospray direct-infusion mass-spectrometry-based metabolomics and lipidomics. Nat Protoc 12:310–328. doi:10.1038/nprot.2016.156. 28079878

[25] Tang K, Page JS, Smith RD. 2004. Charge competition and the linear dynamic range of detection in electrospray ionization mass spectrometry. J Am Soc Mass Spectrom 15:1416–1423. doi:10.1016/j.jasms.2004.04.034. 15465354 PMC1829300

[26] Nothias LF, Petras D, Schmid R, Duhrkop K, Rainer J, Sarvepalli A, Protsyuk I, Ernst M, Tsugawa H, Fleischauer M, Aicheler F, Aksenov AA, Alka O, Allard PM, Barsch A, Cachet X, Caraballo-Rodriguez AM, Da Silva RR, Dang T, Garg N, Gauglitz JM, Gurevich A, Isaac G, Jarmusch AK, Kameník Z, Kang KB, Kessler N, Koester I, Korf A, Le Gouellec A, Ludwig M, Martin HC, McCall LI, McSayles J, Meyer SW, Mohimani H, Morsy M, Moyne O, Neumann S, Neuweger H, Nguyen NH, Nothias-Esposito M, Paolini J, Phelan VV, Pluskal T, Quinn RA, Rogers S, Shrestha B, Tripathi A, van der Hooft JJJ, Vargas F, et al. 2020. Feature-based molecular networking in the GNPS analysis environment. Nat Methods 17:905−908. doi:10.1038/s41592-020-0933-6. 32839597 PMC7885687

[27] Moumbock AFA, Gao M, Qaseem A, Li J, Kirchner PA, Ndingkokhar B, Bekono BD, Simoben CV, Babiaka SB, Malange YI, Sauter F, Zierep P, Ntie-Kang F, Günther S. 2021. StreptomeDB 3.0: an updated compendium of streptomycetes natural products. Nucleic Acids Res 49:D600−D604. doi:10.1093/nar/gkaa868. 33051671 PMC7779017

[28] Okamoto S, Taguchi T, Ochi K, Ichinose K. 2009. Biosynthesis of actinorhodin and related antibiotics: discovery of alternative routes for quinone formation encoded in the act gene cluster. Chem Biol 16:226–236. doi:10.1016/j.chembiol.2009.01.015. 19246012

[29] Miggiels P, Wouters B, van Westen GJP, Dubbelman A-C, Hankemeier T. 2019. Novel technologies for metabolomics: more for less. Trends Anal Chem 120:115323. doi:10.1016/j.trac.2018.11.021.

[30] Zhang X, Romm M, Zheng X, Zink EM, Kim Y-M, Burnum-Johnson KE, Orton DJ, Apffel A, Ibrahim YM, Monroe ME, Moore RJ, Smith JN, Ma J, Renslow RS, Thomas DG, Blackwell AE, Swinford G, Sausen J, Kurulugama RT, Eno N, Darland E, Stafford G, Fjeldsted J, Metz TO, Teeguarden JG, Smith RD, Baker ES. 2016. SPE-IMS-MS: an automated platform for sub-sixty second surveillance of endogenous metabolites and xenobiotics in biofluids. Clin Mass Spectrom 2:1–10. doi:10.1016/j.clinms.2016.11.002. 29276770 PMC5739065

[31] R Development Core Team. 2019. R: a language and environment for statistical computing. R Foundation for Statistical Computing, Vienna, Austria.

[32] Gibb S, Strimmer K. 2012. MALDIquant: a versatile R package for the analysis of mass spectrometry data. Bioinformatics 28:2270–2271. doi:10.1093/bioinformatics/bts447. 22796955

[33] Schiffman C, Petrick L, Perttula K, Yano Y, Carlsson H, Whitehead T, Metayer C, Hayes J, Rappaport S, Dudoit S. 2019. Filtering procedures for untargeted LC-MS metabolomics data. BMC Bioinformatics 20:334. doi:10.1186/s12859-019-2871-9. 31200644 PMC6570933

[34] Franc V, Hlaváč V, Navara M. 2005. Sequential coordinate-wise algorithm for the non-negative least squares problem. Comput Anal Images Patterns 2005:407–414.

[35] Smith CA, Want EJ, O’Maille G, Abagyan R, Siuzdak G. 2006. XCMS: processing mass spectrometry data for metabolite profiling using nonlinear peak alignment, matching, and identification. Anal Chem 78:779–787. doi:10.1021/ac051437y. 16448051

[36] Chambers MC, Maclean B, Burke R, Amodei D, Ruderman DL, Neumann S, Gatto L, Fischer B, Pratt B, Egertson J, Hoff K, Kessner D, Tasman N, Shulman N, Frewen B, Baker TA, Brusniak M-Y, Paulse C, Creasy D, Flashner L, Kani K, Moulding C, Seymour SL, Nuwaysir LM, Lefebvre B, Kuhlmann F, Roark J, Rainer P, Detlev S, Hemenway T, Huhmer A, Langridge J, Connolly B, Chadick T, Holly K, Eckels J, Deutsch EW, Moritz RL, Katz JE, Agus DB, MacCoss M, Tabb DL, Mallick P. 2012. A cross-platform toolkit for mass spectrometry and proteomics. Nat Biotechnol 30:918–920. doi:10.1038/nbt.2377. 23051804 PMC3471674

