# Supplementary figures and images for: SubTap, a Versatile 3D Printed Platform for Eavesdropping on Extracellular Interactions
Source: mSystems. 2021 Aug 24;6(4):e00902-21. doi: 10.1128/mSystems.00902-21 (PMC8422993; doi:10.1128/mSystems.00902-21)

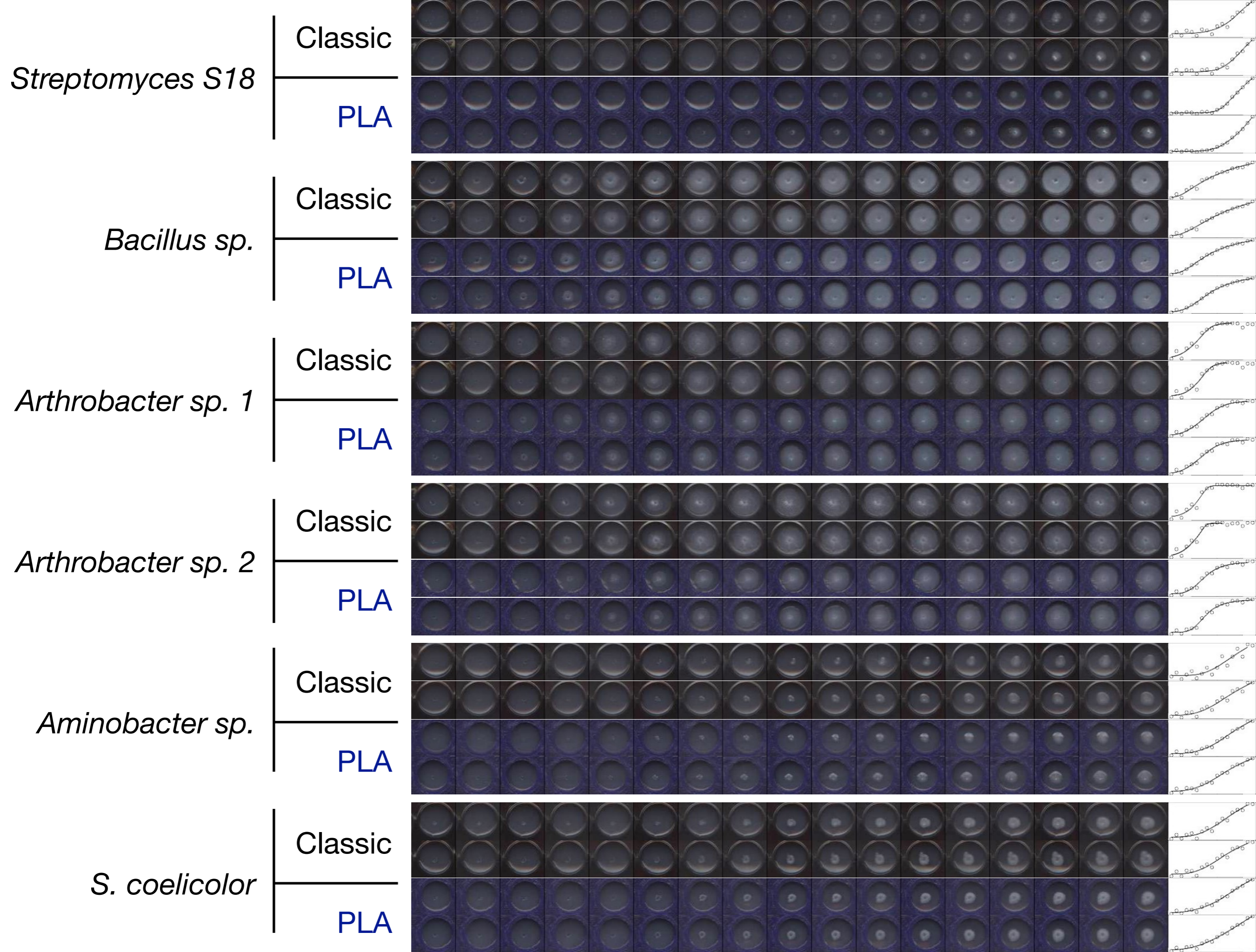

Supplement: FIG S1 [file msystems.00902-21-sf001.pdf]

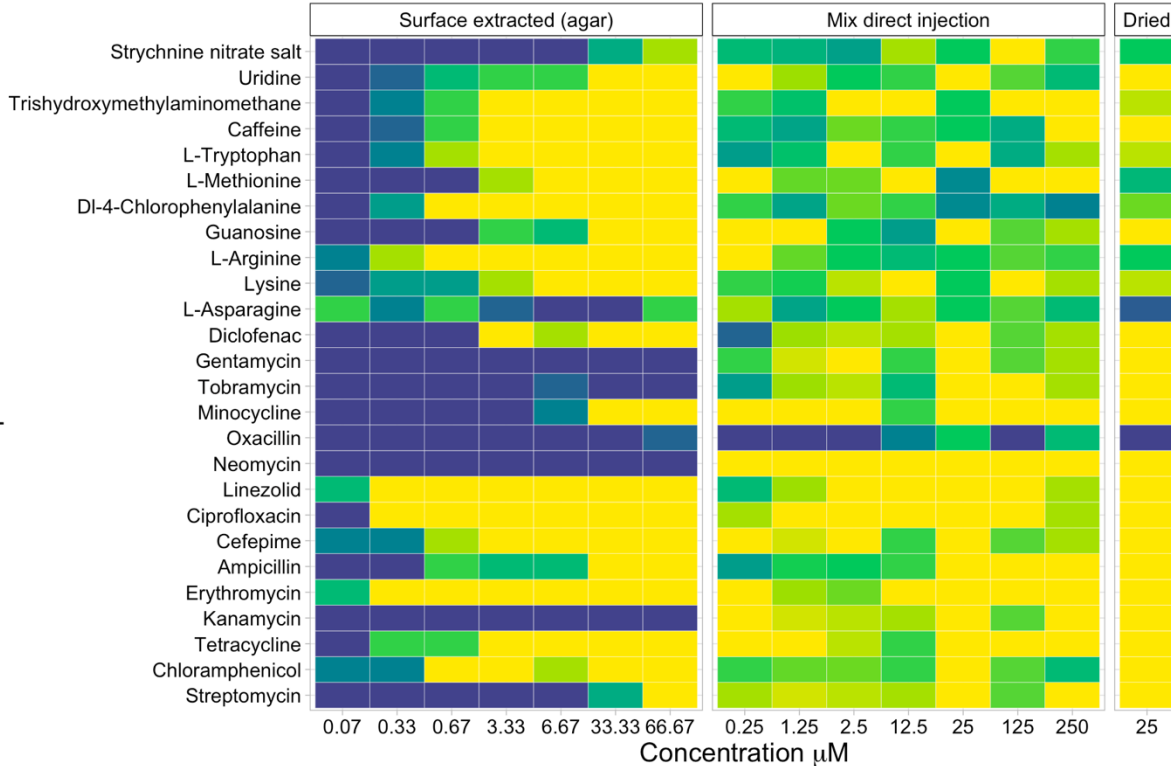

Percentage of detection among replicates

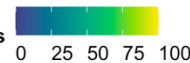

Supplement: FIG S2 [file msystems.00902-21-sf002.pdf]

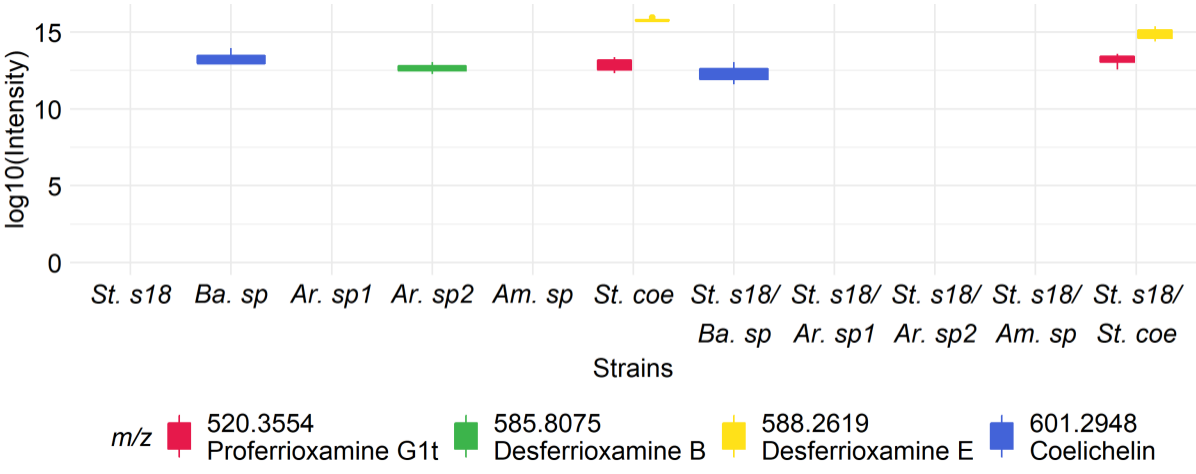

Supplement: FIG S4 [file msystems.00902-21-sf004.pdf]

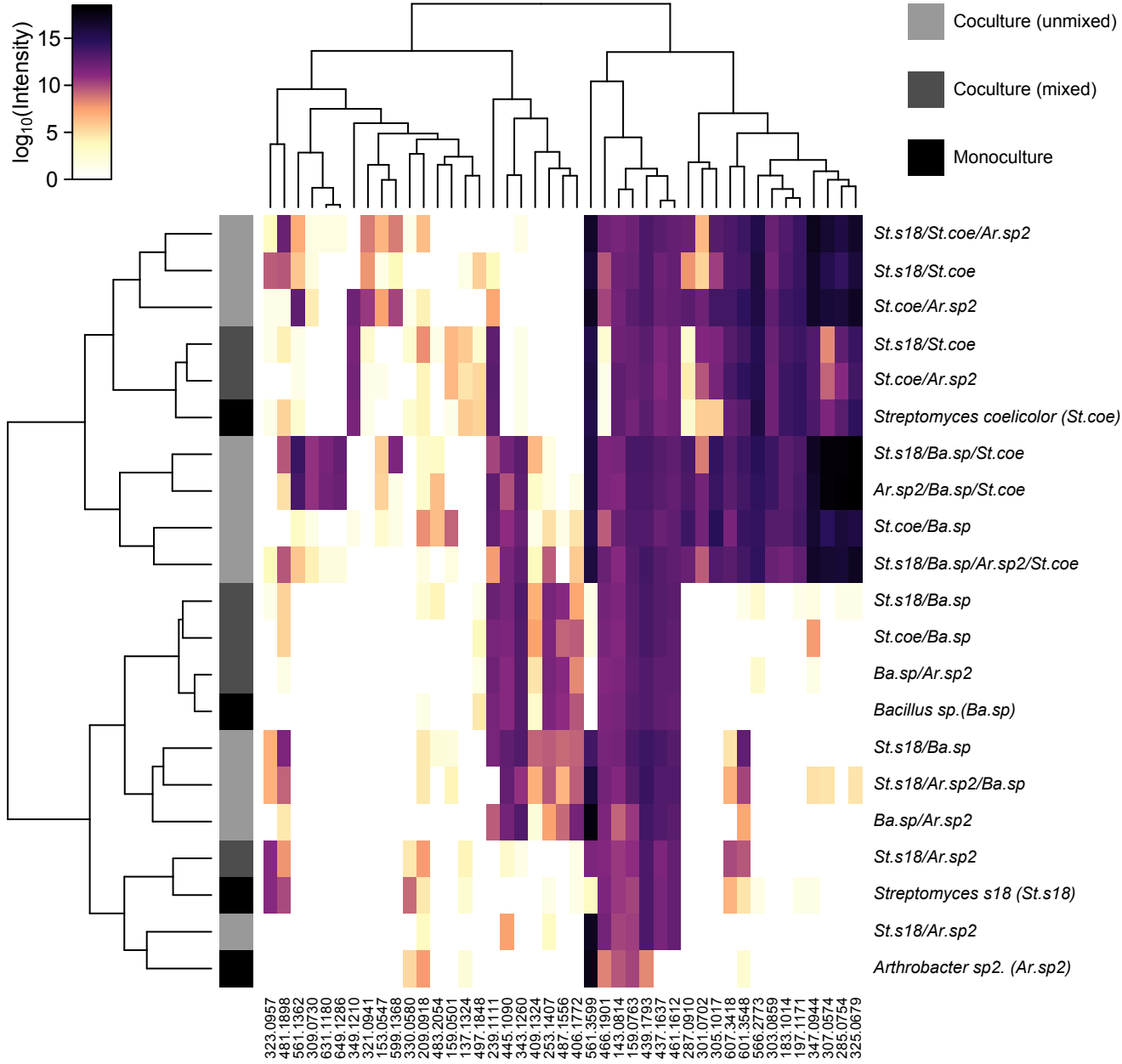

Supplement: FIG S6 [file msystems.00902-21-sf006.pdf]
