# Supplementary material for: SubTap, a Versatile 3D Printed Platform for Eavesdropping on Extracellular Interactions
Source: mSystems. 2021 Aug 24;6(4):e00902-21. doi: 10.1128/mSystems.00902-21 (PMC8422993; doi:10.1128/mSystems.00902-21)
Supplement: TABLE S1 [file msystems.00902-21-st001.pdf]

| **Singly-charged** | **Doubly-charged** | **Triply-charged** |
|---|---|---|
| M+H | M+2H | M+3H |
| M+Na | M+H+Na | M+2H+2Na |
| M+K | M+H+$NH_4$ | M+2Na+H |
| M+$NH_4$ | M+H+K | M+3Na |
| M+$CH_3OH$+H | M+2Na | |
| M+H-$H_2O$ | | |
| M+H-2$H_2O$ | | |
| M+2Na-H | | |
| M+2K-H | | |
| 2M+H | | |
| 2M+Na | | |
| 2M+K | | |
| 2M+$NH_4$ | | |
