# Supplementary material for: SubTap, a Versatile 3D Printed Platform for Eavesdropping on Extracellular Interactions
Source: mSystems. 2021 Aug 24;6(4):e00902-21. doi: 10.1128/mSystems.00902-21 (PMC8422993; doi:10.1128/mSystems.00902-21)
Supplement: TABLE S2 [file msystems.00902-21-st002.pdf]

Name	Molecular formula	Exact mass	Surface extracted (agar)							Mix direct injection							Mix dried and direct injection
			Concentration (μM)														
			66.67	33.33	6.67	3.33	0.67	0.33	0.07	250	125	25	12.5	2.5	1.25	0.25	25
Streptomycin	$C_{21}H_{39}N_7O_{12}$	581.2657	2.29	0.50	0.00	0.00	0.00	0.00	0.00	1.00	0.75	1.00	0.86	0.89	0.92	0.86	1.00
Chloramphenicol	$C_{11}H_{12}Cl_2N_2O_5$	322.0123	2.00	1.50	0.86	1.00	1.00	0.29	0.29	0.57	0.75	1.00	0.71	0.78	0.77	0.71	1.00
Tetracycline	$C_{22}H_{24}N_2O_8$	444.1533	4.00	3.67	1.14	1.14	0.71	0.71	0.00	1.00	1.00	1.00	0.71	0.89	1.00	1.00	1.00
Kanamycin	$C_{18}H_{36}N_4O_{11}$	484.2381	0.00	0.00	0.00	0.00	0.00	0.00	0.00	1.00	0.75	1.00	0.86	0.89	0.92	1.00	1.00
Erythromycin	$C_{37}H_{67}NO_{13}$	733.4612	4.43	4.17	3.29	2.43	1.86	1.71	0.86	1.00	1.00	1.00	1.00	0.78	0.85	1.00	1.00
Ampicillin	$C_{16}H_{19}N_3O_4S$	349.1096	3.43	2.67	0.86	0.86	0.71	0.00	0.00	1.00	1.00	1.00	0.71	0.67	0.69	0.43	1.00
Cefepime	$C_{19}H_{24}N_6O_5S_2$	480.1250	3.29	4.00	2.86	2.57	1.43	0.29	0.29	0.86	0.75	1.00	0.71	1.00	0.92	1.00	1.00
Ciprofloxacin	$C_{17}H_{18}FN_3O_3$	331.1332	2.29	2.17	1.71	1.14	1.00	1.00	0.00	0.86	1.00	1.00	1.00	1.00	1.00	0.86	1.00
Linezolid	$C_{16}H_{20}FN_3O_4$	337.1438	3.71	4.33	2.29	2.43	1.86	1.86	0.57	0.86	1.00	1.00	1.00	1.00	0.85	0.57	1.00
Neomycin	$C_{23}H_{46}N_6O_{13}$	614.3123	0.00	0.00	0.00	0.00	0.00	0.00	0.00	1.00	1.00	1.00	1.00	1.00	1.00	1.00	1.00
Oxacillin	$C_{19}H_{21}N_3O_5S$	403.1202	0.14	0.00	0.00	0.00	0.00	0.00	0.00	0.57	0.00	0.67	0.29	0.00	0.00	0.00	0.00
Minocycline	$C_{23}H_{27}N_3O_7$	457.1849	2.00	2.00	0.29	0.00	0.00	0.00	0.00	1.00	1.00	1.00	0.71	1.00	1.00	1.00	1.00
Tobramycin	$C_{18}H_{37}N_5O_9$	467.2591	0.00	0.00	0.14	0.00	0.00	0.00	0.00	0.86	1.00	1.00	0.57	0.89	0.85	0.43	1.00
Gentamycin	$C_{21}H_{43}N_5O_7$	477.3163	0.00	0.00	0.00	0.00	0.00	0.00	0.00	0.86	0.75	1.00	0.71	1.00	0.92	0.71	1.00
Diclofenac	$C_{14}H_{11}Cl_2NO_2$	295.0167	4.00	3.50	1.86	1.29	0.00	0.00	0.00	0.86	0.75	1.00	0.86	0.89	0.85	0.14	1.00
L-Asparagine	$C_4H_8N_2O_3$	132.0535	0.71	0.00	0.00	0.14	0.71	0.29	0.71	0.57	0.75	0.67	0.86	0.67	0.46	0.86	0.11
Lysine	$C_6H_{14}N_2O_2$	146.1055	3.00	2.33	1.00	0.86	0.43	0.71	0.14	0.86	1.00	0.67	1.00	0.89	0.69	0.71	0.89
L-Arginine	$C_6H_{14}N_4O_2$	174.1117	3.00	2.00	1.00	1.00	1.00	0.86	0.29	0.71	0.75	0.67	0.57	0.67	0.77	1.00	0.67
Guanosine	$C_{10}H_{13}N_5O_5$	283.0917	3.00	3.00	1.14	1.00	0.00	0.00	0.00	0.86	0.75	1.00	0.43	0.67	1.00	1.00	1.00
Dl-4-Chlorophenylalanine	$C_9H_{10}ClNO_2$	199.0400	3.86	3.83	2.29	2.00	1.00	0.43	0.00	0.29	0.50	0.33	0.71	0.78	0.46	0.71	0.78

L-Methionine	$C_5H_{11}NO_2S$	149.0511	3.00	3.00	1.43	1.00	0.00	0.00	0.00	1.00	1.00	0.33	1.00	0.78	0.77	1.00	0.56
L-Tryptophan	$C_{11}H_{12}N_2O_2$	204.0899	3.86	3.83	2.86	2.71	1.14	0.29	0.00	0.86	0.50	1.00	0.71	1.00	0.62	0.43	0.89
Caffeine	$C_8H_{10}N_4O_2$	194.0804	2.14	1.50	1.00	1.00	0.71	0.14	0.00	1.00	0.50	0.67	0.71	0.78	0.46	0.57	1.00
Trishydroxymethyl-aminomethane	$C_4H_{11}NO_3$	121.0739	2.43	2.33	1.86	1.57	0.86	0.43	0.00	1.00	1.00	0.67	1.00	1.00	0.62	0.71	0.89
Uridine	$C_9H_{12}N_2O_6$	244.0695	2.00	2.17	1.14	1.14	0.57	0.14	0.00	0.57	0.75	1.00	0.71	0.67	0.85	1.00	1.00
Strychnine nitrate salt	$C_{21}H_{23}N_3O_5$	397.1638	0.86	0.50	0.00	0.00	0.00	0.00	0.00	0.71	1.00	0.67	0.86	0.44	0.54	0.57	0.67
