# Supplementary material for: SubTap, a Versatile 3D Printed Platform for Eavesdropping on Extracellular Interactions
Source: mSystems. 2021 Aug 24;6(4):e00902-21. doi: 10.1128/mSystems.00902-21 (PMC8422993; doi:10.1128/mSystems.00902-21)
Supplement: FIG S3 [file msystems.00902-21-sf003.pdf]

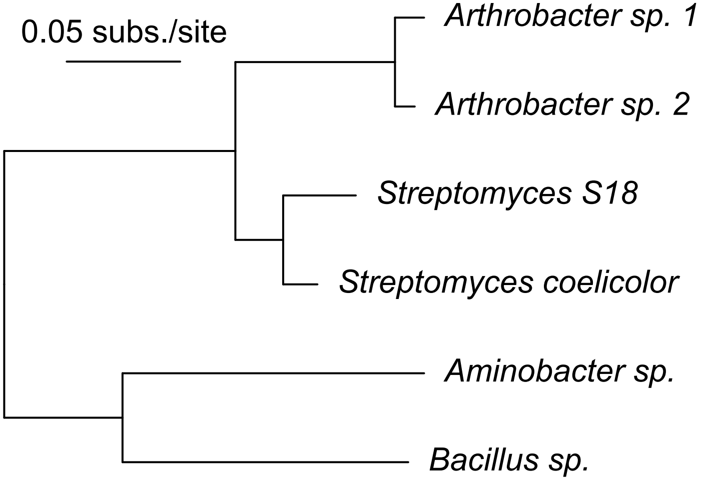
0.05 subs./site
Arthrobacter sp. 1
Arthrobacter sp. 2
Streptomyces S18
Streptomyces coelicolor
Aminobacter sp.
Bacillus sp.
