# Supplementary material for: SubTap, a Versatile 3D Printed Platform for Eavesdropping on Extracellular Interactions
Source: mSystems. 2021 Aug 24;6(4):e00902-21. doi: 10.1128/mSystems.00902-21 (PMC8422993; doi:10.1128/mSystems.00902-21)
Supplement: TABLE S3 [file msystems.00902-21-st003.pdf]

m/z Found	Detected	Molecule	Adduct	MQScore	Shared Peaks
147.0450	St. s18/St. coe	Glutamate	M+H	0.72	6.00
147.0960	Am. sp	4-Guanidinobutanoate	M+H	0.84	6.00
153.0430	Ar. sp1; Ar. sp2	6-N-Hydroxylaminopurine	M+H	0.83	6.00
154.0590	Ar. sp2; St. s18/Ar. sp2	Benzoic acid. 2.3-dihydroxy	M+H	0.80	6.29
162.0960	Ba. sp; St. s18/Ba. sp	alpha.-Methyl cinnamic acid	M+H	0.72	6.00
164.1080	St. coe; St. s18/St. coe	3-(2-Hydroxyphenyl)propanoate	M+H	0.79	6.00
166.1230	St. coe	L-Ethionine	M+H	0.75	6.00
169.0840	Ba. sp; St. s18/Ba. sp	Lupinine	M+H	0.83	6.00
173.1650	Am. sp; St. s18/Am. sp	L-Arginine	M+H	0.90	6.00
175.0370	Ar. sp2; St. s18/Ar. sp2	L-Arginine	M+H	0.72	6.13
184.9800	Ar. sp2; St. s18/Ar. sp2	cyclo-(Ala-4-hydroxy-Pro)	M+H	0.74	6.00
189.1070	Am. sp	N.alpha.-Acetyl-L-lysine	M+H	0.71	6.00
191.1550	St. coe	alpha.-Cyano-4-hydroxycinnamic acid	M+H	0.78	6.00
194.9460	Ar. sp2	10H-Acridin-9-one	M+H	0.71	6.00
197.1180	St. coe; St. s18/St. coe	1-Hydroxyphenazine	M+H	0.75	6.25
201.0550	St. coe; St. s18/St. coe	Leu-Ser	M+H-H_2O	0.83	6.00
205.0510	Ba. sp; St. s18/Ba. sp	DL-Thioctamide	M+H	0.71	6.00
206.1140	Am. sp; St. s18/Am. sp	2-(1-hydroxyethyl)-4-(2-hydroxypropyl)-2H-furan-5-one	M+NH_4	0.72	6.00
210.1490	St. coe; St. s18/St. coe	Kainic Acid	M-H	0.72	6.00
213.0470	Am. sp	Pro-Pro	M+H	0.76	6.00
222.1130	St. coe; St. s18/St. coe	Zeatin	M+H	0.73	6.00
224.1650	St. coe	Phenazine-1-carboxylic acid	M+H	0.75	6.00
226.1450	St. coe; St. s18/St. coe	L-Carnosine	M+H	0.86	6.40
227.0350	St. s18/St. coe	PyroGlu-Pro	M+H	0.78	6.00
234.1360	St. coe	His-Pro	M+H-H_2O	0.95	9.00
238.1790	Ba. sp; St. s18/Ba. sp	Pyrrolizidine alkaloid A	M+H	0.77	6.00
241.1000	St. coe	O-Succinyl-L-homoserine	M+Na	0.85	6.09
242.1400	St. coe	Asn-Gln	M+H-NH_3	0.73	6.00
243.1580	Am. sp	PyroGlu-Ile	M+H	0.80	7.00
248.2090	Ba. sp; St. s18/Ba. sp	gamma-Glutamylvaline	M+H	0.78	6.41
250.7010	Am. sp; St. s18/Am. sp	His-Ile	M+H-H_2O	0.79	8.32
252.0620	St. coe	Leu-His	M+H-H_2O	0.71	8.42
253.1280	St. coe	Hydroxy-spiciferinone	M+H	0.70	7.67
258.9130	Ar. sp2	Ile-Lys	M+H	0.75	9.57
259.0270	Am. sp; St. s18/Am. sp	Ile-Lys	M+H	0.74	11.44
261.1200	Am. sp	Oxolinic Acid	M+H	0.73	6.00
261.1610	St. coe	Flumequine	M+H	0.74	6.40
262.1170	Ba. sp; St. s18/Ba. sp	Lys-Ile	$M+H^+$	0.71	10.22
273.1820	Ba. sp; St. s18/Ba. sp	Sotalol	M+H	0.76	6.00
274.0930	Ba. sp; St. s18/Ba. sp	Epiandrosterone	M-H_2O+H	0.74	6.00
275.0330	St. coe; St. s18/St. coe	Val-Arg	M+H	0.78	7.88

m/z Found	Detected	Molecule	Adduct	MQScore	Shared Peaks
275.0960	Ba. sp; St. s18/Ba. sp	Val-Arg	M+H	0.71	6.00
277.1560	St. coe	L-Saccharopine	M+H	0.72	6.40
281.0800	St. s18/St. coe	Dihydrotanshinone I	M+H	0.70	6.00
287.0840	St. coe; St. s18/St. coe	2-nonenyl-3-hydroxy 4(1H)-quinolone ! 4-hydroxy-2-nonenylquinoline N-oxide	M+H	0.73	8.00
291.1710	St. coe	3.7.8-trihydroxy-3-methyl-10-oxo-1.4-dihydropyrano[4.3-b]chromene-9-carboxylic acid	M-H_2O+H	0.80	6.00
296.1110	Ba. sp; St. s18/Ba. sp	Acaranoic acid	M+H	0.75	6.44
299.0470	Ar. sp1; Am. Sp	S-Adenosyl-L-methionine	M+H	0.77	8.00
299.0650	Ba. sp; St. s18/Ba. sp	Cryptotanshinone	M+H	0.73	6.25
302.1030	St. coe; St. s18/St. coe	Aleuretic Acid	M+H	0.74	6.00
305.1030	St. coe; St. s18/St. coe	Arachidonoyl amide	M+H	0.80	6.30
305.1390	Am. sp	2-Methylene-5-(2.5-Dioxotetrahydrofuran-3-Yl)-6-Oxo--10.10-Dimethylbicyclo[7: 2: 0]Undecane	M-H	0.71	6.00
307.0590	St. coe; St. s18/St. coe	(3S)-8-hydroxy-3-methyl-3.4-dihydro-2H-benzo[a]anthracene-1.7.12-trione	M+H	0.70	6.00
311.1380	Ba. sp; St. s18/Ba. sp	trans-EKODE-(E)-Ib	M-H	0.71	7.00
327.0848	St. coe; St. s18/St. coe	Leuconolam	M+H	0.71	6.00
387.1390	Ba. sp; St. s18/Ba. sp	(3R.5S.7R.9S.10S.12S.13R.14S.17R)-10.13-dimethyl-17-((R)-6-methylheptan-2-yl)hexadecahydro-1H-cyclopenta[a]phenanthrene-3.7.12-triol	M-2H_2O+H	0.74	10.46
429.1540	Am. Sp	Leupeptin	M+H	0.71	10.00
520.3550	St. coe; St. s18/St. coe	Proferrioxamine G1t	M+H	0.71	13.50
585.8080	Ar. sp2	Desferrioxamine B + Al (M-2H+Al)	M+H	0.72	15.00
588.2620	St. coe; St. s18/St. coe	Coelichelin	M+Na	0.83	17.76
601.2950	Ba. sp; St. s18/Ba. sp	Desferrioxamine E	M+H	0.82	17.00
653.192	St. coe St. s18/St. coe	Polysaccharide Agarose x4	M+Na	0.80	14.87
