# Supplementary material for: SubTap, a Versatile 3D Printed Platform for Eavesdropping on Extracellular Interactions
Source: mSystems. 2021 Aug 24;6(4):e00902-21. doi: 10.1128/mSystems.00902-21 (PMC8422993; doi:10.1128/mSystems.00902-21)
Supplement: FIG S5 [file msystems.00902-21-sf005.pdf]

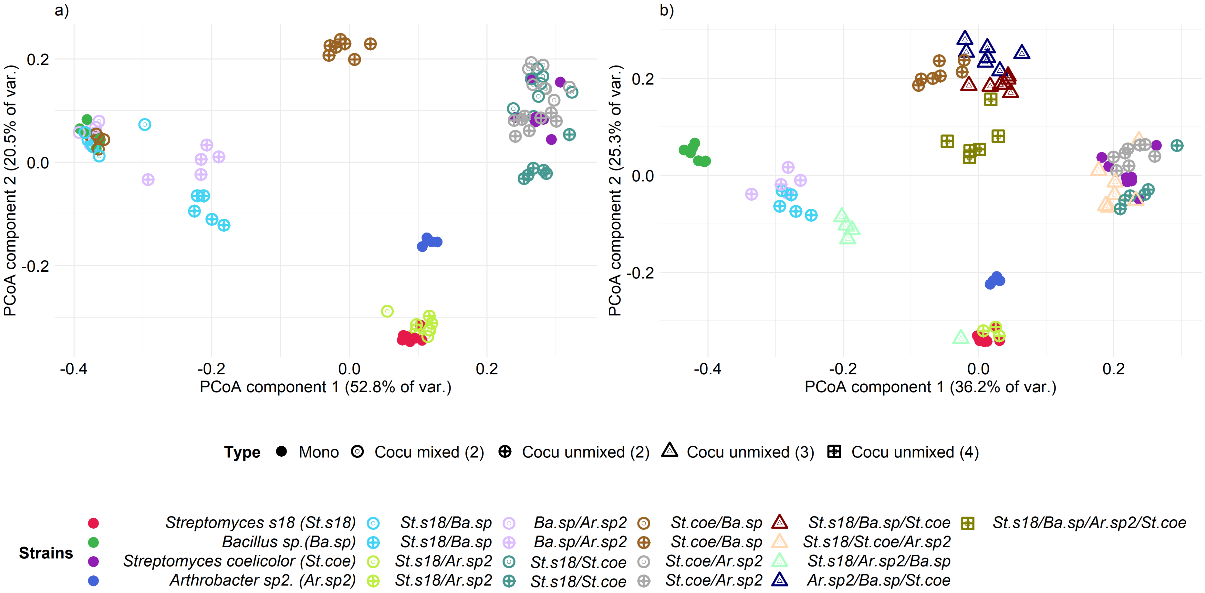

a)
b)
PCoA component 1 (52.8% of var.)
PCoA component 2 (20.5% of var.)
PCoA component 1 (36.2% of var.)
PCoA component 2 (25.3% of var.)
0.2
0.0
-0.2
-0.4
Type
Mono
Cocu mixed (2)
Cocu unmixed (2)
Cocu unmixed (3)
Cocu unmixed (4)
Strains
Streptomyces s18 (St.s18)
Bacillus sp.(Ba.sp)
Streptomyces coelicolor (St.coe)
Arthrobacter sp2. (Ar.sp2)
St.s18/Ba.sp
St.s18/Ba.sp
St.s18/Ar.sp2
St.s18/Ar.sp2
Ba.sp/Ar.sp2
Ba.sp/Ar.sp2
St.s18/St.coe
St.s18/St.coe
St.coe/Ba.sp
St.coe/Ba.sp
St.coe/Ar.sp2
St.coe/Ar.sp2
St.s18/Ba.sp/St.coe
St.s18/St.coe/Ar.sp2
St.s18/Ar.sp2/Ba.sp
Ar.sp2/Ba.sp/St.coe
St.s18/Ba.sp/Ar.sp2/St.coe
