# Supplementary material for: SubTap, a Versatile 3D Printed Platform for Eavesdropping on Extracellular Interactions
Source: mSystems. 2021 Aug 24;6(4):e00902-21. doi: 10.1128/mSystems.00902-21 (PMC8422993; doi:10.1128/mSystems.00902-21)
Supplement: TABLE S4 [file msystems.00902-21-st004.pdf]

Compound name	m/z Found [M+H]$^+$	Mass error (ppm)
9.10-Octalin	137.1324	4.52
Ectoin	143.0815	3.85
6-Methylsalicylic acid	153.0547	2.63
Allantoin	159.0502	10.36
Hydroxyectoine	159.0764	3.53
Germicidin B	183.1015	3.41
Germicidin	197.1171	3.04
Kynurenine	209.0919	3.65
(R)-3.5-Diethyl-4-hydroxy-5-((E)-2-methylbuta-1.3-dienyl)thiophen-2(5H)-one	239.1112	2.32
CHEMBL3087384	253.1407	13.08
CHEMBL1242196	285.0755	2.90
(S)-DNPA	287.0911	2.96
Kalafungin A	301.0703	3.16
Dihydrokalafungin	303.0859	2.92
T3HN	305.1017	2.68
Kinobscurinone	307.0575	10.32
Fluostatin K	309.0731	10.53
DHK-OH	321.0942	10.20
3 Methyltetraphene 1.6.7.8.12 pentol	323.0958	11.96
Fluostatin C	325.0679	10.14
Cyclic AMP	330.0580	6.92
Disaccharide (e.g., Trehalose)	343.1260	5.82
Wailupemycin G	347.0945	7.30
Coelimycin P1	349.1211	3.25
Roseoflavin	406.1773	11.49
Fluostatin J	409.1325	9.19
Fluostatin L	437.1637	8.41
3'-N-Demethyl-4'-hydroxystaurosporine	439.1793	5.27
Granaticin	445.1091	9.75
Oxytetracycline	461.1612	11.24
Brasiliquinone A	466.1902	7.62
Staurosporine M1	481.1899	4.70
3'-N-Acetylholyrine A	483.2055	4.61
Mycothiol	487.1557	8.36
9-Hydroxyl-3'-N-acetyl-4'-Hydroxylstaurosporine	497.1848	4.58
CHEMBL372196	561.1362	6.23
Deferoxamine	561.3600	2.26
2-[[2-Amino-5-[formyl(hydroxy)amino]pentanoyl]amino]-5-[[2-[[2-amino-5-[formyl(hydroxy)amino]pentanoyl]amino]-3-hydroxybutanoyl]-hydroxyamino]pentanoic acid	566.2774	2.21
Difluostatin A	599.1368	4.37
Nocardamin	601.3549	2.06
Tautomycetin	607.3418	10.51
gamma-Actinorhodin	631.1180	14.59
epsilon-Actinorhodin	649.1286	14.31
